# Management of Chordoma and Chondrosarcoma with Fractionated Stereotactic Radiotherapy

**DOI:** 10.3389/fsurg.2017.00035

**Published:** 2017-06-23

**Authors:** Harish N. Vasudevan, David R. Raleigh, Julian Johnson, Adam A. Garsa, Philip V. Theodosopoulos, Manish K. Aghi, Christopher Ames, Michael W. McDermott, Igor J. Barani, Steve E. Braunstein

**Affiliations:** ^1^Department of Radiation Oncology, University of California San Francisco, San Francisco, CA, United States; ^2^Department of Radiation Oncology, Icahn School of Medicine at Mount Sinai, New York, NY, United States; ^3^Department of Neurological Surgery, University of California San Francisco, San Francisco, CA, United States

**Keywords:** chordoma, chondrosarcoma, fractionated stereotactic radiotherapy, CyberKnife, stereotactic body radiotherapy

## Abstract

**Objective:**

To evaluate the efficacy and toxicity of fractionated stereotactic radiotherapy (FSRT) for chordoma and chondrosarcoma.

**Methods:**

Twenty consecutive patients with a histopathologic diagnosis of chordoma (*n* = 16) or chondrosarcoma (*n* = 4) treated between 2010 and 2016 were retrospectively identified. All patients underwent FSRT in five fractions to a median dose of 37.5 Gy (range: 25–40 Gy) and followed with serial magnetic resonance imaging. Overall survival (OS), local recurrence-free survival (LRFS), and event-free survival (EFS) were estimated using the Kaplan–Meier method.

**Results:**

With a median follow-up of 28 months after FSRT and 40 months after initial surgery, crude OS and LRFS were 90%. Nine patients (45%) reported grade 1–3 acute toxicity, and two patients (10%) experienced grade 4, 5 late toxicity. One patient previously treated with proton therapy died from radiation vasculopathy 9 months after FSRT. The use of FSRT for recurrent disease or in patients with prior radiation therapy was associated with significantly decreased EFS.

**Conclusion:**

FSRT for chordoma and chondrosarcoma is associated with high rates of OS and local control. Although many patients experience acute toxicity, there is a low incidence of late toxicity or irreversible treatment related morbidity despite the frequency of prior radiotherapy in this population. FSRT is an effective adjuvant or salvage treatment for chordoma and chondrosarcoma.

## Introduction

Chordomas are rare, locally aggressive tumors developing from remnants of the embryonic notochord predominantly within the skull base, vertebral spine, and sacralcoccygeal region (1, 2). Similarly, chondrosarcomas are slow growing tumors that commonly arise in the skull base or vertebral spine that tend to recur locally (3). Given their propensity for particular anatomical locations and high risk of local recurrence, chordomas and chondrosarcomas historically share management approaches, although emerging evidence suggests chordomas are clinically more aggressive than chondrosarcomas (4–8). The standard of care dictates maximal safe surgical resection with radiation therapy to reduce the risk of local recurrence both following gross total resection and in anatomical settings, such as the skull base, where complete resection is not feasible (5, 9–11). Indeed, multiple studies have noted the challenge in achieving total surgical resection of these tumors without significant morbidity due to their predilection to invade critical structures (11–14). Thus, optimizing the efficacy of radiation therapy for chordoma and chondrosarcoma represents a critical step in improving patient outcomes.

Given their tendency for local recurrence despite aggressive measures, chordomas are classically considered radioresistant, and accordingly, early studies of radiation therapy for chordoma prescribed large doses in the range of 60–75 Gy over a period of 5–7 weeks (9, 15, 16). However, the lack of a clear dose–response relationship coupled with the adverse effects associated with such doses motivated investigation of alternatives to conventionally fractionated high-dose photon therapy (17), with recent efforts focusing on the application of charged particles such as protons (18, 19), or hypofractionated stereotactic approaches (12, 18, 20–22) for the treatment of chordomas and chondrosarcomas. Although the improved outcomes associated with radiation therapy are well established, the optimal radiation treatment modality, dose, and fractionation regimen remains incompletely understood.

Here, we report a series of 20 consecutive patients with chordoma or chondrosarcoma from a single institution treated with fractionated stereotactic radiotherapy (FSRT). Advantages of a stereotactic approach for these tumors include augmented biologic effective dose, highly conformal treatment fields, and steep dose reduction outside target volumes (21–23). Further, the limited availability of proton facilities and shorter treatment times with hypofractionated courses makes FSRT logistically attractive. In the present study, we analyze overall survival (OS), local recurrence-free survival (LRFS), and toxicity profiles following FSRT. We find high rates of OS and local control with low incidence of long-term morbidity, supporting the utility of FSRT for chordoma and chondrosarcoma. All patient information has been de-identified, and all patients consented to the publication of their de-identified data.

## Materials and Methods

### Patient Population

A total of 20 consecutive patients (12 males, 8 females) with a histopathologic diagnosis of either chordoma (*n* = 16, 80%) or chondrosarcoma (*n* = 4, 20%) who were treated between 2010 and 2016 at a single institution were retrospectively identified (Table 1). All patients were treated with FSRT. Demographic and clinical data were extracted from the medical record and institutional cancer registry. The extent of tumor resection was determined from postoperative magnetic resonance imaging (MRI), and all included patients were following with serial MRIs after completion of FSRT. This study was approved by the institutional review board (#15-16205).

**Table 1:** Clinical characteristics and fractionated stereotactic radiotherapy (FSRT) usage in chordoma and chondrosarcoma patients.

Parameter	All locations (*n* = 20)	Skull base (*n* = 12)	Sacrum (n = 5)	Spine (*n* = 3)
Age (years)	45 (15–76)	37.5 (15–62)	64 (34–71)	68 (38–76)
Male sex	12 (60%)	9 (75%)	2 (40%)	1 (33%)
Female sex	8 (40%)	3 (25%)	3 (60%)	2 (67%)
Histological type	–	–	–	–
Chordoma	16 (80%)	9 (75%)	4 (80%)	3 (100%)
Chondrosarcoma	4 (20%)	3 (25%)	1 (20%)	0
Surgical resection	18 (90%)	11 (92%)	5 (100%)	2 (67%)
Gross total resection	10 (56%)	3 (27%)	5 (100%)	2 (100%)
Subtotal resection	8 (44%)	8 (73%)	0	0
Preoperative FSRT	4 (20%)	0	3 (60%)	1 (33%)
Postoperative FSRT	14 (70%)	11 (92%)	2 (40%)	1 (33%)
Surgery to FSRT interval (months)	4 (2–145)	2.5 (2–145)	16, 49	10
Definitive FSRT	2 (10%)	1 (8%)	0	1 (33%)
Upfront FSRT	15 (75%)	10 (83%)	3 (60%)	2 (67%)
Recurrent FSRT	5 (25%)	2 (17%)	2 (40%)	1 (33%)
Reirradiation	6 (30%)	3 (25%)	2 (40%)	1 (33%)

*Only those patients receiving FSRT with follow-up posttreatment imaging were included in our analysis (*n* = 20). All six reirradiated patients are included in the postoperative FSRT group, and five of these patients were treated for recurrent disease. Data are represented as median (range) or absolute number of patients (%)*.

The median patient age at diagnosis was 45 years (range: 15–76 years). Of the 20 tumors, 12 (60%) were located in the skull base (9 in the clivus), 5 (20%) in the sacrum, and 3 (15%) in the spine. The median age at diagnosis was 45 years old (range 15–76). Typical presenting symptoms included vision changes (*n* = 8 or 67% of skull base cases), sacral pain (*n* = 5 or 100% of sacrum cases), and back pain (*n* = 3 or 100% of spine cases). Eighteen patients (90%) underwent surgical resection, of which 10 patients (50%) were reported to have a gross total resection. The majority of patients (*n* = 14, 70%) received FSRT postoperatively, and the median interval between surgery and FSRT for these patients was 4 months (range: 2–145 months). The use of FSRT in the postoperative setting was commonly observed for skull base lesions (*n* = 11, 93%) while preoperative FSRT (*n* = 4) was only reported for sacral (*n* = 3, 60%) and spine (*n* = 1, 33%) tumors. In addition, most patients underwent FSRT as part of initial therapy (*n* = 15, 75%), again particularly for lesions location in the skull base (*n* = 10, 83%). FSRT was used for recurrent disease in five patients (25%) or in patients with a prior history of radiation (*n* = 6, 30%). Clinical and imaging outcomes were assessed over a median follow-up period of 28 months (range: 4.4–70 months) from time of FSRT and 40 months (range: 7.1–188 months) from time of histopathologic diagnosis at resection or biopsy.

### FSRT Parameters

All patients underwent a simulation computed tomography (CT) scan and diagnostic MRI for radiation treatment planning with the Multiplan system (Accuray, Inc., Sunnyvale, CA, USA). For intracranial or cervical spine tumors, patients were immobilized with a thermoplastic mask; individualized vacuum bags were used to immobilize patients for thoracic, lumbar, and sacral treatment. The gross tumor volume (GTV) and critical structures (such as the brain, brainstem, optic nerves, and spinal cord) were delineated on CT-MRI fusions by the treating radiation oncologist. For patients treated with preoperative or definitive FSRT, the clinical target volume (CTV) was equivalent to the GTV except in the case of spine tumors, in which the CTV was expanded to include the entire vertebral body. For patients receiving postoperative FSRT, the CTV included any residual gross disease and regions at-risk for local recurrence, such as bone or soft tissue. The planning target volume (PTV) was generated by adding a 1- to 3-mm isotropic margin to the CTV. When necessary, PTV coverage with the prescription dose was sacrificed to respect normal tissue tolerance as specified by TG101. During treatment, patient position was typically monitored every 30–60 s, and for patients with spinal or sacral lesions, XSight (Accuray, Inc., Sunnyvale, CA, USA) spine tracking was used. FSRT was delivered either every day or every alternate day, Monday–Friday, at the discretion of the treating physician.

The median FSRT dose was 37.5 Gy (range: 25–40 Gy), and all patients received five fractions (Table 2). The median prescription isodose was 62.5% (range: 52–93%), with a median maximum dose of 53.90 Gy (range: 38–68 Gy). The median PTV varied with anatomic location from a minimum of 14.75 cm^3^ (range: 7.1–37 cm^3^) in the skull base, 265 cm^3^ (range: 102–424 cm^3^) in the sacrum, and 226.23 cm^3^ (range: 114–314 cm^3^) in the vertebral spine. Critical structure dose constraints were also defined relative to tumor location. For skull base tumors, the median optic nerve D_max_ was 16.79 Gy (range: 1.1–28 Gy), the median chiasm D_max_ was 15.98 Gy (range: 0.93–27 Gy), and the median brainstem D_max_ was 28.84 Gy (range: 23–33 Gy). For spine tumors, the median spinal cord D_max_ was 29.43 Gy (range: 21–32 Gy).

**Table 2:** Fractionated stereotactic radiotherapy (FSRT) dosimetry details and toxicity for chordoma and chondrosarcoma patients.

Parameter	All locations (*n* = 20)	Skull base (*n* = 12)	Sacrum (*n* = 5)	Spine (*n* = 3)
FSRT dose (Gy)	37.5 (25–40)	35 (25–40)	40 (30–40)	40 (25–40)
Prescription isodose	62.5% (52–93%)	62% (52–92%)	69% (63–93%)	62% (61–62%)
Dmax (Gy)	53.90 (38–68)	53.90 (38–68)	46.88 (38–63)	64.52 (40–66)
Gross tumor volume (cm^3^)	18.95 (0.65–414)	10.29 (0.65–22)	90.34 (75–414)	128.00 (49–276)
Planning target volume (cm^3^)	36.50 (7.07–424)	14.75 (7.1–37)	265.00 (102–424)	226.23 (114–314)
Dmax optic nerve (Gy)	–	16.79 (1.1–28)	–	–
Dmax chiasm (Gy)	–	15.98 (0.93–27)	–	–
Dmax brainstem (Gy)	–	28.84 (23–33)	–	–
Dmax spinal cord (Gy)	–	–	–	29.43 (21–32)
Acute toxicity	9 (45%)	5 (42%)	2 (40%)	2 (67%)
Cranial neuropathy	3 (15%)	3 (24%)	0	0
Peripheral neuropathy	2 (10%)	0	1 (20%)	1 (33%)
Mucositis	2 (10%)	0	0	2 (67%)
Other	–	Hearing loss (2)	Wound infection (1)	–
		Headache (2)		
Late toxicity	2 (10%)	2 (17%)	0	0

*All patients received five fractions. Acute toxicity was defined as occurring within 3 months of FSRT treatment completion, and many patients reported multiple toxicities. Both cases of late toxicity involved radiation necrosis, and grade 5 radiation vasculopathy was reported in one of these patients with a prior history of proton therapy. Note both patients who experienced late toxicity also experienced acute toxicity. Data are represented as median (range) or absolute number of patients (%)*.

### Clinical Evaluation Criteria

For follow-up, patients underwent clinical examination and MRI every 3–6 months immediately following completion of treatment and then annually after 1–3 years. Local control was defined as no increase in tumor size on surveillance imaging. Toxicity was retrospectively collected, with acute toxicity defined as occurring within 3 months of FSRT completion, and reported using the Common Terminology Criteria for Adverse Events (CTCAEv4.0) (Table 2). Follow-up imaging was also evaluated for evidence of radiation necrosis or other radiographic features consistent with radiation toxicity.

### Statistical Analysis

The Kaplan–Meier method was used to estimate OS, LRFS, and event-free survival (EFS), the latter of which was defined as local recurrence, regional recurrence, distant recurrence, or death. Analysis was conducted from both the date of FSRT and the date of initial surgery or biopsy corresponding to the time of histopathologic diagnosis. The Mantel–Cox (log-rank test) was used to compare differences in survival among patient subgroups. A two-sided *p*-value < 0.05 was considered statistically significant. All plots were generated in GraphPad Prism (GraphPad Software, La Jolla, CA, USA), and analysis was carried out in both GraphPad and R v3.2.4 (https://www.r-project.org/).

## Results

### OS and LRFS in Chordoma and Chondrosarcoma Patients Treated with FSRT

**Figure 1:**
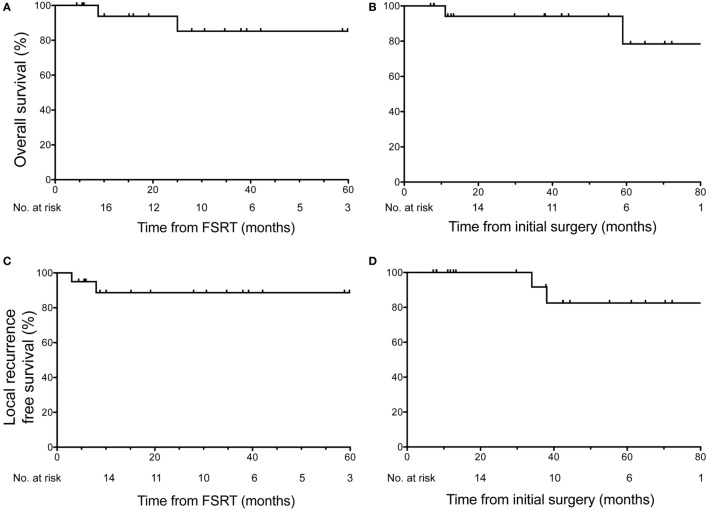
Overall survival (OS) and local recurrence free survival in all patients. OS across all 20 patients shown relative to **(A)** time of fractionated stereotactic radiotherapy (FSRT) and **(B)** time of initial surgery or biopsy. Two deaths were reported during follow-up, the first 9 months after FSRT (11 months after surgery) for recurrent clival chordoma and the second 25 months after FSRT (59 months after surgery) in a patient with recurrent sacral chordoma. Local recurrence-free survival across all 20 patients shown relative to **(C)** time of FSRT and **(D)** time of initial surgery or biopsy revealed two local recurrences in the follow-up period. Median follow-up from time of FSRT was 28 months, and median follow-up from time of initial surgery/biopsy was 40 months.

At the time of last clinical follow-up, 90% of patients (*n* = 18) were alive. From time of FSRT, the median follow-up was 28 months, and the estimated OS for all patients was 93.8% at 1 year (95% CI: 63–99%) and 85.23% at 3 years (95% CI: 52–96%) (Figure 1A). From time of initial surgery, the median follow-up was 40 months, and the estimated OS for all patients was 94.12% at 1 year (95% CI: 65–99%), and 78.43% at 5 years (95% CI: 32–95%) (Figure 1B). Two deaths were reported during the follow-up period. One patient died from radiation vasculopathy 9 months after receiving FSRT to a total dose of 40 Gy in five fractions that was performed postoperatively 2 months following surgery for recurrent clival chordoma. This patient previously received proton therapy to 70 Gy 28 months prior to time of death (19 months prior to FSRT). The second mortality occurred 25 months after FSRT to a total dose of 30 Gy in five fractions that was performed postoperatively 34 months after resection of a multiply recurrent sacral chordoma previously treated with proton therapy and two courses of FSRT. In this patient, the cause of death was cardiovascular disease likely unrelated to chordoma progression or radiation toxicity.

With regard to LRFS, 90% of patients (*n* = 18) had no local recurrence at the time of last assessment. From the time of FSRT, the estimated LRFS rate for all patients was 88.67% at 3 years (95% CI: 61–97%) (Figure 1C). From the time of initial surgery, the estimated LRFS rate for all patients was 91.67% at 3 years (95% CI: 54–99%) and 82.50% at 5 years (95% CI: 46–95%) (Figure 1D). Two local recurrences were reported in the follow-up period, both within the first year following FSRT. The first local recurrence occurred 3 months after receiving FSRT to a total dose of 40 Gy in five fractions that was performed 31 months after resection of a recurrent cervical vertebral chordoma. This patient subsequently developed additional radiographic evidence of recurrence, first within the cervical spine at 3 months as described above and again distantly in submental nodules after 21 months. Both recurrences were retreated with salvage FSRT and at last follow-up, 24 months after initial FSRT, this patient was disease free. The second local recurrence occurred 8 months after FSRT to 30 Gy in five fractions (38 months after surgery) for a multiply recurrent sacral chordoma, who was subsequently retreated with FSRT. As described above, this patient ultimately died of cardiovascular disease 25 months after initial FSRT.

### Decreased EFS in Patients with Recurrent Disease and Prior History of Radiation

**Figure 2:**
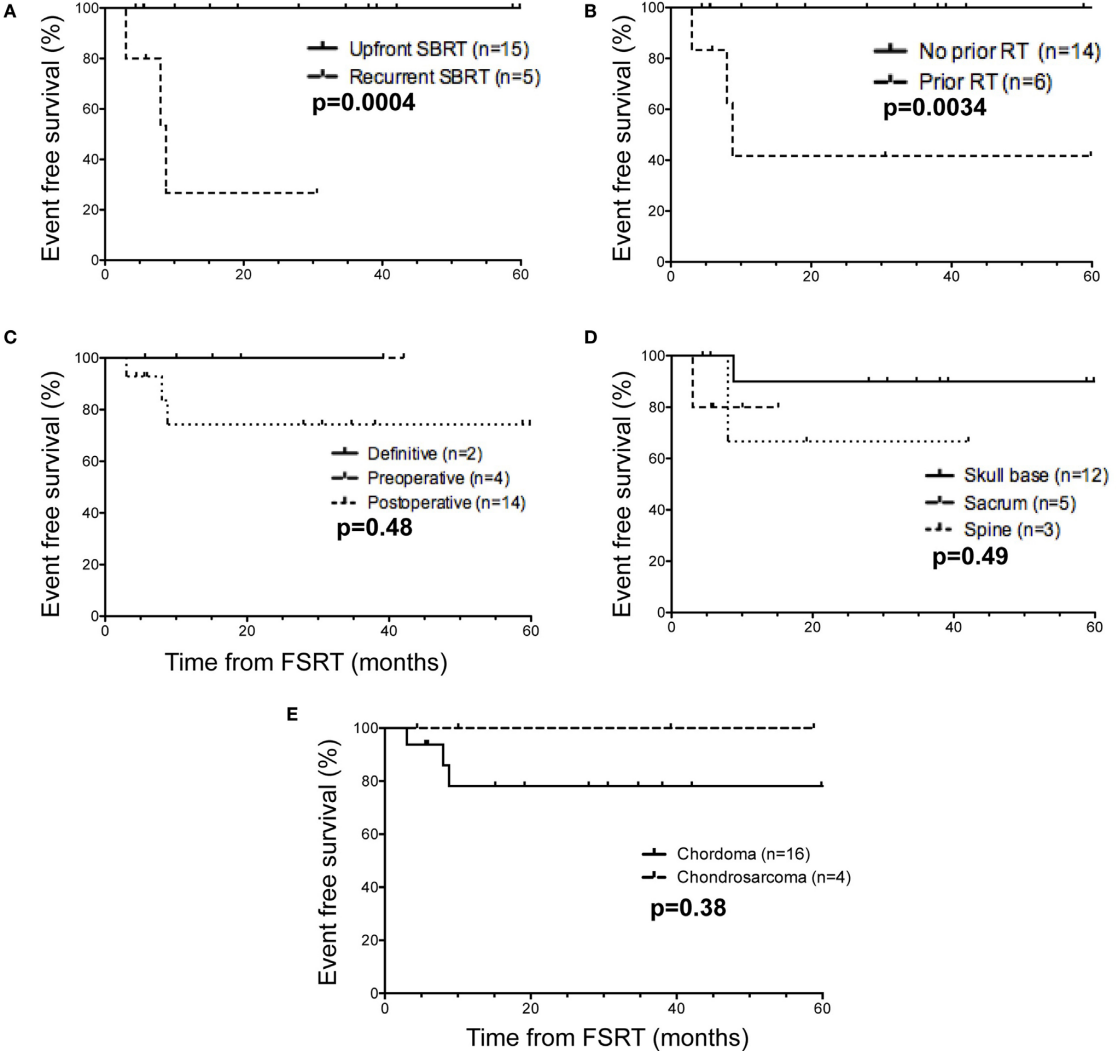
Event-free survival in selected patient subgroups relative to time of fractionated stereotactic radiotherapy (FSRT). **(A)** When separating FSRT in the upfront (solid line) versus recurrent (dashed line) setting, a minority of patients (*n* = 5) received FSRT for recurrent disease, and this group encompassed all patients with significant events (3/5). **(B)** Considering prior history of radiation therapy (protons or EBRT) (*n* = 6), all patients with reported events (3/6) received radiation therapy before undergoing FSRT. **(C)** With respect to definitive (solid line) versus preoperative (dashed line) versus postoperative FSRT (dotted line), the majority of patients (*n* = 14) received FSRT in the postoperative setting, including all patients with significant events (4/14). **(D)** Based on anatomic site of the tumor, one local recurrence was in a sacral tumor (dashed line), and the second recurrence was in a tumor in the cervical spine (dotted line). For skull base tumors (black), one death but no local recurrences were observed. **(E)** With regard to histological subtype, the majority of patients were diagnosed with chordoma (*n* = 16, black line) compared to chondrosarcoma (*n* = 4, dashed), and all three events occurred in chordoma patients. Significant events were defined as tumor recurrence (local, regional, or distant) or death, and reported *p*-values are from the log-rank (Mantel–Cox) test.

Given the level of patient heterogeneity in the population receiving FSRT for chordoma or chondrosarcoma, several patient subgroups were explored to identify settings in which FSRT may be particularly efficacious. When considering the use of upfront FSRT compared to treatment for recurrent disease, the former patients show significantly improved EFS (*p* = 0.0004) (Figure 2A). Indeed, all significant events occurred in patients treated for recurrent disease within 1 year following FSRT completion. With regard to past history of radiation therapy, patients who did not receive radiation prior to undergoing FSRT exhibited significantly improved EFS compared to those with a prior history of radiotherapy (*p* = 0.0034) (Figure 2B). All significant events occurred in the subset of patients who were previously treated with radiation; it is important to note all five patients treated with FSRT in the recurrent setting also had a prior history of radiation, underscoring the overlap between these two patient subpopulations. Specifically, three patients received FSRT for recurrence following adjuvant proton therapy, one received FSRT for a recurrence following adjuvant external beam radiation therapy, one received multiple courses of FSRT for recurrent disease, and one received FSRT immediately following postoperative external beam radiation therapy due to presence of gross residual disease.

The majority of patients received FSRT postoperatively (*n* = 14), and there were no significant differences in EFS compared to preoperative (*n* = 4) or definitive FSRT (*n* = 2) (Figure 2C). For patients treated with preoperative FSRT (*n* = 4), the median interval from FSRT to surgery was 4 weeks (range: 2–6 weeks), and pathology in one case showed evidence of tumor necrosis. None of these patients had documented recurrence in the follow-up period (range: 7–44 months). Further, there was no significant difference in EFS with respect to anatomical location when comparing patients with skull base tumors (*n* = 12), sacral tumors (*n* = 5), or spine tumors (*n* = 3) (Figure 2D). Given the more indolent course of chondrosarcoma compared to chordoma, we next compared outcomes between chordoma (*n* = 16) and chondrosarcoma (*n* = 4). Although all three patients with significant events were in the chordoma subgroup, no significant difference in EFS was observed between the chordoma and chondrosarcoma histopathological subgroups (Figure 2E), likely due in part to the high proportion of chordomas compared to chondrosarcomas in our series.

### Treatment-Associated Toxicity following FSRT

Acute toxicity (*n* = 9, 45% of all patients) occurring within 3 months of FSRT completion was more commonly reported than late toxicity (*n* = 2, 10% of all patients) (Table 2). Acute side effects were dependent on tumor location and included grade 1–3 cranial neuropathy (*n* = 3, 24% of skull base tumors), hearing loss (*n* = 2, 17% of skull base tumors), grade 2 headache (*n* = 2, 17% of skull base tumors), grade 2, 3 peripheral neuropathy (*n* = 2, 25% of sacral and spine tumors), grade 2, 3 mucositis (*n* = 2, 67% of spine tumors), and grade 3 wound infection (*n* = 1, 20% of sacral tumors). All acute toxicities resolved spontaneously within 6 months except for one patient with clival chordoma who had persistent mild hearing loss on long-term follow-up that first presented following completion of FSRT.

Two patients experienced significant late toxicities from treatment. As discussed previously, one patient developed grade 5 radiation vasculopathy 9 months after SBRT for a clival chordoma that was previously treated with proton therapy to the same location 28 months prior. This patient also received surgical re-resection, and he was the only patient in our series to undergo salvage reoperation for recurrent disease. The second patient with late toxicity originally presented with hearing loss due to a chordoma arising from the right jugular foramen and subsequently underwent a subtotal resection followed by adjuvant FSRT to a total dose of 40 Gy in five fractions This patient later developed radiographic, asymptomatic radiation necrosis in the pons apparent on follow-up MRI 37 months after completion of FSRT. At the most recent clinic visit 38 months after completion of FSRT, the patient noted persistent but stable right ear deafness and no other focal neurologic deficits.

## Discussion

In the present study, we report a series of 20 consecutive patients with chordoma or chondrosarcoma treated with FSRT across a range of settings and anatomical locations. With a median follow-up of 40 months after initial surgery, we find 90% of patients (*n* = 18) were alive without evidence of recurrent disease, leading to high rates of OS (85.23% at 3 years after FSRT) and LRFS (91.67% at 3 years after FSRT). Further, our data suggest less durable outcomes for patients undergoing FSRT in the recurrent setting or following prior radiation therapy. Notably, all observed events occurred in patients with recurrent disease who previously received proton therapy, highlighting the increased risk associated with disease recurrence and multiple courses of radiation as well as the possibility of a significantly radioresistant tumor subset that does not respond to standard radiation treatment approaches. Finally, only 10% of patients (*n* = 2) reported long-term side effects, one of whom received prior proton therapy that likely contributed toward the development of radiation vasculopathy.

Our study has a number of limitations, including the small number of patients, low incidence of reported events, and relatively short follow-up period. The small sample size particularly affects the ability to identify patient subgroups based on either clinical parameters or histological subtype that are more likely to benefit from FSRT or exhibit a higher risk for treatment-related toxicity. The median follow-up of 40 months after surgery likely explains, in part, the low incidence of adverse events, and continued long-term follow-up will be essential to build upon the trends observed in the present work. In the interim, the data presented here may serve as the foundation for future prospective trials of FSRT for chordoma and chondrosarcoma, which will be essential given the additional intrinsic limitations of all single-institution retrospective studies such as the work presented here, including selection bias, institutional bias, information bias, and the potential presence of unknown confounders.

The management of chordomas and chondrosarcomas remains challenging. Surgical resection remains the cornerstone for achieving good outcomes in patients with these tumors, but the optimal adjuvant treatment paradigm to insure long-term recurrence-free survival remains unknown. In addition, while both chordoma and chondrosarcoma are often managed similarly, chordomas are increasingly appreciated to be clinically more aggressive than chondrosarcomas, which may motivate the development of distinct treatment approaches. Although high-dose proton therapy provides 72–80% OS and 60–81% local control at 5 years with low toxicity and sparing of critical structures (18, 19), the limited availability of these facilities remains an obstacle to such an approach being widely adopted as a standard of care. Similarly, the use of image-guided intensity-modulated radiation therapy has comparable efficacy, with 86–88% OS and 65–88% local control at 5 years with differences between chordomas and chondrosarcomas, again underscoring the difference in clinical behavior and prognosis between these histologic subtypes; however, the need for daily CT scans over a total of 40 fractions may again limit the widespread adoption of such a regimen (24). Thus, the importance of exploring hypofractionated stereotactic radiation therapy as a treatment modality is become increasingly apparent.

Multiple studies have assessed single fraction stereotactic radiosurgery for chordoma and chondrosarcoma management, but FSRT has theoretical advantages compared to single fraction treatment including a lower risk of radiation toxicity in nearby critical structures and the possibility of safely treating larger tumor volumes with multiple fractions (20, 22, 23, 25–30). A limited number of published series evaluate FSRT for chordomas and chondrosarcomas (10, 23, 31–33), and our study provides additional evidence regarding the efficacy and toxicity associated with its use. Previous work from the North American Gamma Knife Consortium retrospectively examining single fraction treatment demonstrated a 5-year OS of 80% with an improved 5-year OS of 93% in patients with no prior RT (28), consistent with outcomes observed in our data. With regard to fractionated stereotactic approaches such as CyberKnife, retrospective studies have reported similar results. One study of 18 chordoma patients reported an OS of 74% and local control of 59% at 5 years (32), while another study of 20 chordoma patients demonstrated an OS of 53% at 5 years (33). Our outcomes are consistent, and perhaps superior to these results, as we found 94.12% OS and 91.67% LRFS at 3 years following initial surgery. However, it is important to emphasize that comparisons between these retrospective studies are inherently limited, especially as heterogeneity in patient characteristics between and within each series can significantly affect outcomes. Tumor histology, tumor size, quality of surgical resection, interval between surgery and RT, dose coverage, prior radiation therapy, and recurrent disease are all important parameters influencing local control and survival.

## Conclusion

Fractionated stereotactic radiotherapy for chordoma and chondrosarcoma is feasible, well tolerated, and provides rates of OS and LRFS comparable to published data for other treatment modalities. Patients with recurrent disease or previously treated with radiation therapy may be more likely to have decreased rates of local control and OS. Toxicities are primarily acute and resolve spontaneously in most cases, with long-term adverse effects more likely in the small number of patients who received prior radiation therapy. Although sample size and follow-up period are limited, these data suggest FSRT is an effective treatment modality for chordoma and chondrosarcoma, and in the absence of prospective randomized data, provides evidence supporting the use of FSRT in these patients.

## Ethics Statement

This study was carried out in accordance with the recommendations of UCSF institutional review board with written informed consent from all subjects. All subjects gave written informed consent in accordance with the Declaration of Helsinki. The protocol was approved by the UCSF institutional review board, protocol #15-6205.

## Author Contributions

Research design and conception: HV, DR, AG, IB, and SB. Data assembly and analysis: HV, DR, JJ, AG, IB, and SB. Manuscript and figure preparation: HV, DR, and SB. Critical oversight and review: all.

## Conflict of Interest Statement

The authors report no conflict of interest concerning the materials or methods used in this study or the findings specified in this paper.
